# Quantification of mast cells in central centrifugal cicatricial alopecia

**DOI:** 10.1016/j.jdin.2023.11.008

**Published:** 2023-12-18

**Authors:** Sydney Look-Why, Jared Goldberg, Claire Alexanian, Nicole Rogers, Nikita N.M. Coleman, Yolanda M. Lenzy, Eric Pinos, Lynne J. Goldberg

**Affiliations:** a Boston University Chobanian and Avedisian School of Medicine, Boston, Massachusetts; b Consumer Edge, New York, New York; c Department of Dermatology, Boston Medical Center, Boston, Massachusetts; d Tulane University School of Medicine, New Orleans, Louisiana; e International University of the Health Sciences, Basseterre, Saint Kitts and Nevis; f Lenzy Dermatology, Chicopee, Massachusetts; g University of Connecticut School of Medicine, Farmington, Connecticut; h University of Massachusetts, Amherst, Massachusetts

**Keywords:** alopecia, central centrifugal cicatricial alopecia, fibrosis, mast cells, scarring alopecia, skin of color

## Abstract

**Background:**

Mast cells (MCs) have recently been implicated in lymphocytic scarring alopecias, which may share a common pathogenesis. MCs in central centrifugal cicatricial alopecia (CCCA) have not been studied.

**Objective:**

We looked for the presence of MCs in CCCA using 2 different stains to see if their numbers correlated with the number of hair follicles, the degree of inflammation and perifollicular fibrosis, disease duration and severity, and patient symptoms.

**Methods:**

We performed a retrospective review of biopsies of patients diagnosed with CCCA, tabulated MC counts and correlated them with histopathologic and clinical findings.

**Results:**

MC counts were significantly greater using immunoperoxidase staining with CD117 than Giemsa stain, and more were present when the isthmus level was included with the infundibulum. MC counts with CD117 immunostain significantly correlated with the degree of inflammation. MC counts with both stains were significantly associated with the degree of fibrosis independently and after controlling for other factors.

**Limitations:**

The study was limited by insufficient tissue remaining in a small number of the transversely cut blocks.

**Conclusion:**

Our findings may have therapeutic implications for CCCA and other types of lymphocytic scarring alopecia.

**Capsule Summary**

• A common mast cell signature has been found in lymphocytic scarring alopecias. This study quantifies mast cell numbers in central centrifugal cicatricial alopecia.
• The finding that mast cell number correlates with degree of perifollicular fibrosis in central centrifugal cicatricial alopecia suggests that mast cell inhibition may potentially play a therapeutic role.

## Introduction

Primary cicatricial (scarring) alopecias are a group of inflammatory disorders characterized by significant and irreversible damage to the hair follicle (HF) stem cells at the bulge region of the HF. These conditions result in damage to the pilosebaceous unit of the HF, perifollicular fibrosis, and eventual progressive hair loss. They are divided by the predominant inflammatory cell type on histology into lymphocytic, neutrophilic, and mixed varieties,^1^ with the lymphocytic scarring alopecias being more common. The 3 major lymphocytic scarring alopecias are lichen planopilaris (LPP), its variant frontal fibrosing alopecia, and central centrifugal cicatricial alopecia (CCCA).

CCCA is a chronic, progressive form of primary cicatricial alopecia that affects women of African descent. This condition typically starts in the third or fourth decade, presenting with alopecia and often pruritus of the crown or vertex scalp that slowly progresses centrifugally.^2^ Although hair care practices are often implicated in the development of CCCA,^3^ more recent studies suggest a role of autosomal dominant inheritance,^4^ and Malki et al^5^ reported that 24% of patients with CCCA harbor the *PADI3* gene. Histopathologic findings include premature desquamation of the inner root sheath and compound HFs with perifollicular fibrosis and/or inflammation.^6^ Although there are no US Food and Drug Administration approved treatments, antiinflammatory agents including topical and intralesional corticosteroids, topical calcineurin inhibitors, doxycycline, and hydroxychloroquine are often used. The main goals of treatment are symptomatic relief and halting progression of the disease, as once the disease is established regrowth of hair is unlikely.

Mast cells (MCs) are known to reside in the skin and other tissues where they play a regulatory role under normal conditions, however they can be proinflammatory under pathologic conditions.^7^ Their presence has been found to be increased in nonscarring alopecias including telogen effluvium, androgenetic alopecia, and alopecia areata in comparison to control skin.^8^, ^9^, ^10^ Several more recent studies have implicated the presence of MCs in the pathogenesis of scarring alopecia.^11^, ^12^, ^13^ Giemsa has been the stain most commonly used to identify MCs, although 2 studies used toluidine blue.^1^^,^^12^

MCs have been previously studied in LPP, which has both clinical and histopathologic overlap with CCCA. Hobo et al^11^ described a large number of MCs in biopsies of LPP, hypothesizing a possible pathogenic role. Wang et al^12^ found a shared MC gene signature among the lymphocytic scarring alopecias, which in turn prompted clinical use of antihistamines therapeutically.^14^ Thus, we sought to understand the role that MCs play in CCCA. Our primary aim was to quantify the number of MCs and evaluate for an association with both clinical features including disease duration, disease severity, and disease symptoms, and histopathologic findings such as the number of remaining HFs, the degree of inflammation, and the degree of perifollicular fibrosis. Our secondary aim was to compare the usefulness of immunohistochemical staining and standard histochemical staining for MCs.

## Methods

All scalp biopsies taken by 3 providers that were interpreted as CCCA by the Skin Pathology Laboratory at Boston University between January 1, 2015, and December 30, 2021 were identified, and the tissue blocks were retrieved. One hundred sequential specimens (from 98 patients) with sufficient remaining tissue in the block (based on visual inspection) were included in the study. Three slides were cut from each block and stained with hematoxylin and eosin, Giemsa stain, and immunoperoxidase stain for CD117 (staining done at Applied Pathology Systems).

For each specimen, the deepest level present on transverse section (infundibulum or isthmus), the number of terminal and vellus HFs, the degree of perifollicular inflammation, and the degree of perifollicular fibrosis was recorded. The latter 2 were graded on a 4-point scale relative to the normal thickness of follicular epithelium as follows: none = 1; mild (defined as less than the radial diameter) = 2; moderate (defined as equal to the radial diameter) = 3; and heavy (defined as greater than the radial diameter) = 4. For each specimen, the 3 high power fields with the most MCs were identified and counted simultaneously by 3 of the authors (SLY, CA, and LJG). Cases which lacked 3 high power fields were excluded. To standardize counting, only MCs with a nucleus in the plane of section were recorded. Cell counts were consolidated by averaging across the 3 individuals, and this aggregated count was used for analysis.

For each patient, relevant clinical data were retrieved from the medical record. The following information was identified and recorded: patient age, duration of disease, disease severity (classification proposed by Olsen et al^15^), and the presence and degree of symptoms.

Several statistical analyses were performed. To assess interrater reliability in counting MCs, the intraclass correlation coefficient was computed using an average rater 2-way random effect model. Paired samples *t* test was used to compare MC counts between the Giemsa and CD117 stains. Welch’s unpaired samples *t* test was used to analyze differences in MC counts between the levels of the section and between patients who had and did not have symptoms. Pearson correlation was used to analyze the relationship between MC counts and the number of total and terminal HFs, the degree of inflammation and perifollicular fibrosis, and patient characteristics including age, duration of disease, and disease severity. Multiple linear regression was used to control for factors while further analyzing the relationship between MC counts and perifollicular fibrosis. Two patients lacked disease duration data and were excluded from duration analysis. All statistical analyses were performed using R version 3.6.3 (R Core Team 2020). Two-tailed *P* < .05 indicated significance.

## Results

Patients ranged in age from 24 to 85 years with a mean of 48 years. The duration of disease had a broad range, from newly diagnosed to a 21 year history of disease. Most patients (69%) had early disease (stages 1 and 2), and 31% had advanced disease (stage 3).

Out of the 100 specimen blocks selected, after cutting 13 had insufficient tissue for counting of 3 high power fields and were excluded. Two patients had 2 biopsies. Of the 87 biopsies from 85 patients that were suitable, 55 included only the infundibulum level and 32 extended to the isthmus. There was excellent interrater agreement for both the Giemsa stain (ICC_112_ = 0.991, *P* < .001) and CD117 (ICC_193_ = 0.995, *P* < .001).

The total number of HFs ranged from 2 to 28 with a median of 11. The number of terminal HFs ranged from 1 to 28 to with a median of 7. The number of vellus HFs ranged from 0 to 15 with a median of 2. In most patients terminal HFs outnumbered vellus HFs, 5 patients displayed equal numbers, and in 9 patients vellus HFs outnumbered terminal HFs.

**Fig 1:**
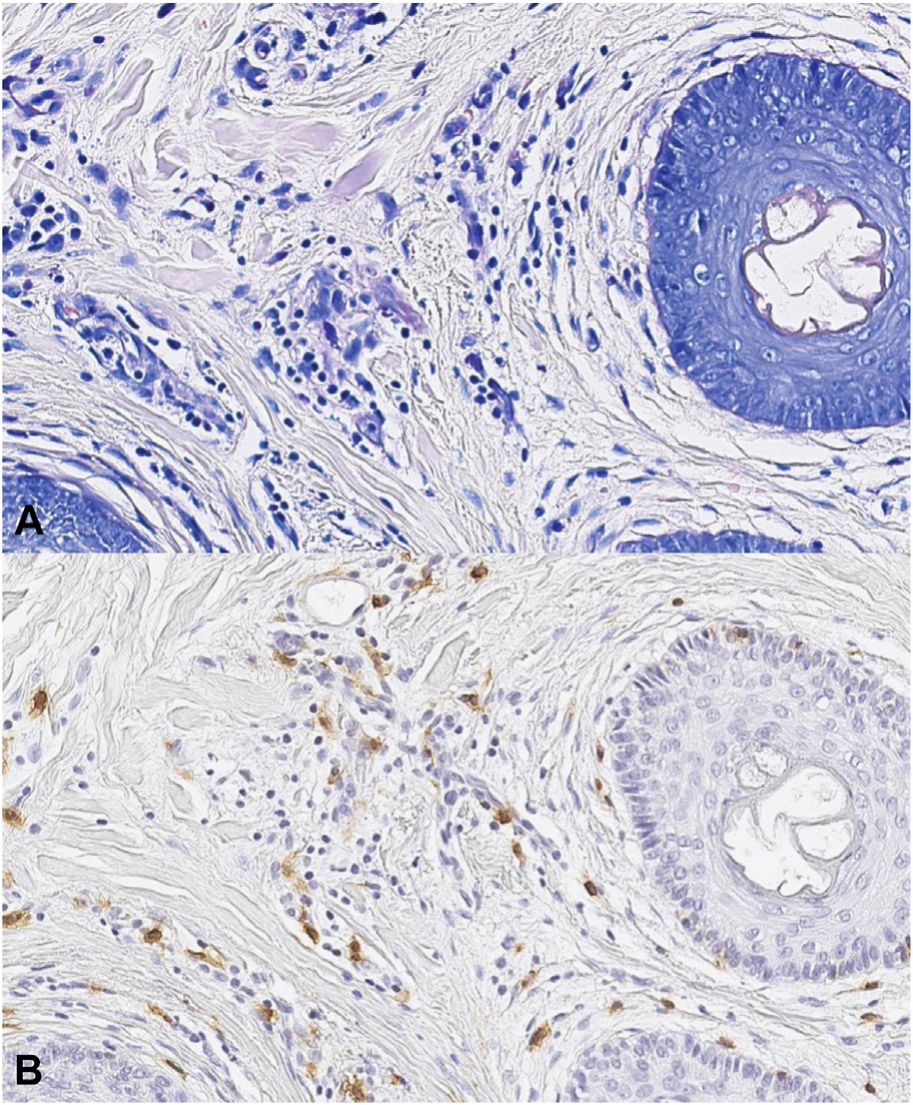
Mast cells in central centrifugal cicatricial alopecia visualized with **(A)** Giemsa stain and **(B)** immunoperoxidase stain for CD117.

**Fig 2:**
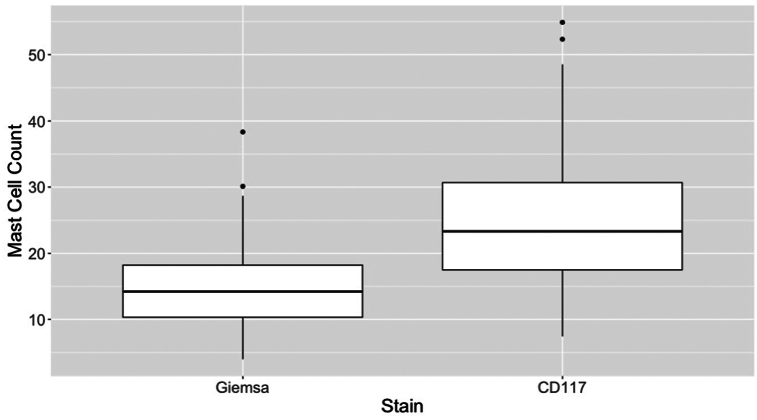
Central centrifugal cicatricial alopecia mast cell count distribution by stain. There were significantly more mast cells detected with immunoperoxidase stain for CD117, *P* < .001.

MC numbers varied depending on the stain being used. The average number of MCs detected by Giemsa stain ranged from 4 to 38 with a median of 14. The average number of MCs detected by immunoperoxidase staining with CD117 ranged from 7 to 55 with a median of 23. A sample of the staining is shown in Fig 1. Immunoperoxidase staining for CD117 demonstrated significantly more MCs than were detected by Giemsa stain, with a mean difference of 9.7 cells per section and a standard deviation of 7.04 (*t*_86_ = 12.9, *P* < .001, Fig 2).

**Fig 3:**
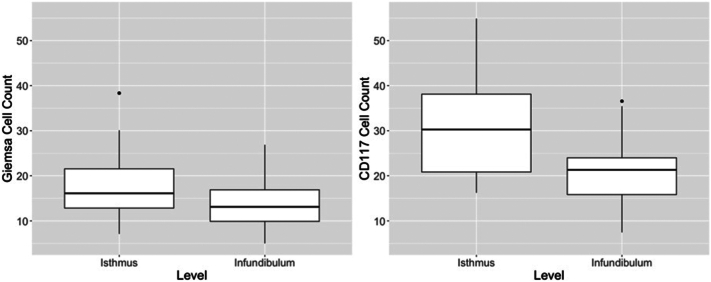
Central centrifugal cicatricial alopecia mast cell count distributions with Giemsa stain and immunoperoxidase stain for CD117 at the levels of the isthmus and infundibulum. Biopsies containing the isthmus demonstrated significantly more mast cells (Giemsa *P* = .01, CD117 *P* < .001).

**Fig 4:**
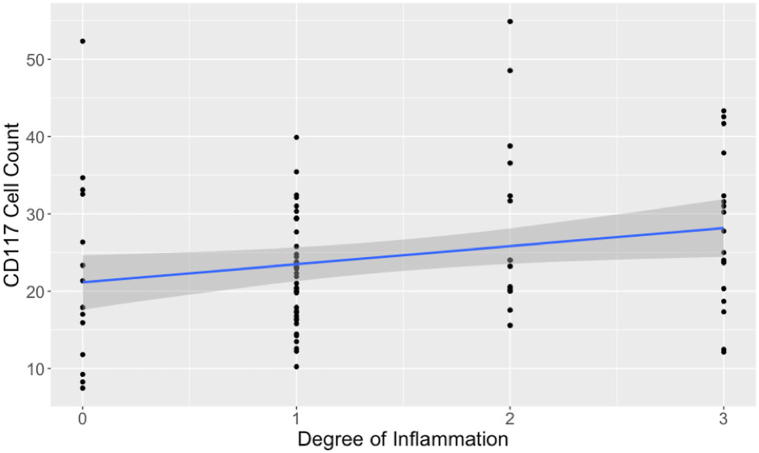
Central centrifugal cicatricial alopecia mast cell counts with immunoperoxidase stain for CD117 significantly correlated with the degree of inflammation, *P* = .023.

MC numbers also varied with the plane of section and the degree of inflammation. Significantly more MCs were present in specimens that extended to the isthmus for both stains (Giemsa: *t*_47.9_ = –2.65, *P* = .01; CD117: *t*_48.2_ = –4.34, *P* < .001, Fig 3). MC counts positively correlated with the degree of inflammation, but this reached statistical significance only for CD117 (*t*_85_ = 2.32, *P* = .023, Fig 4). There was no difference in MC counts between location of maximum inflammation (perifollicular vs perivascular).

**Fig 5:**
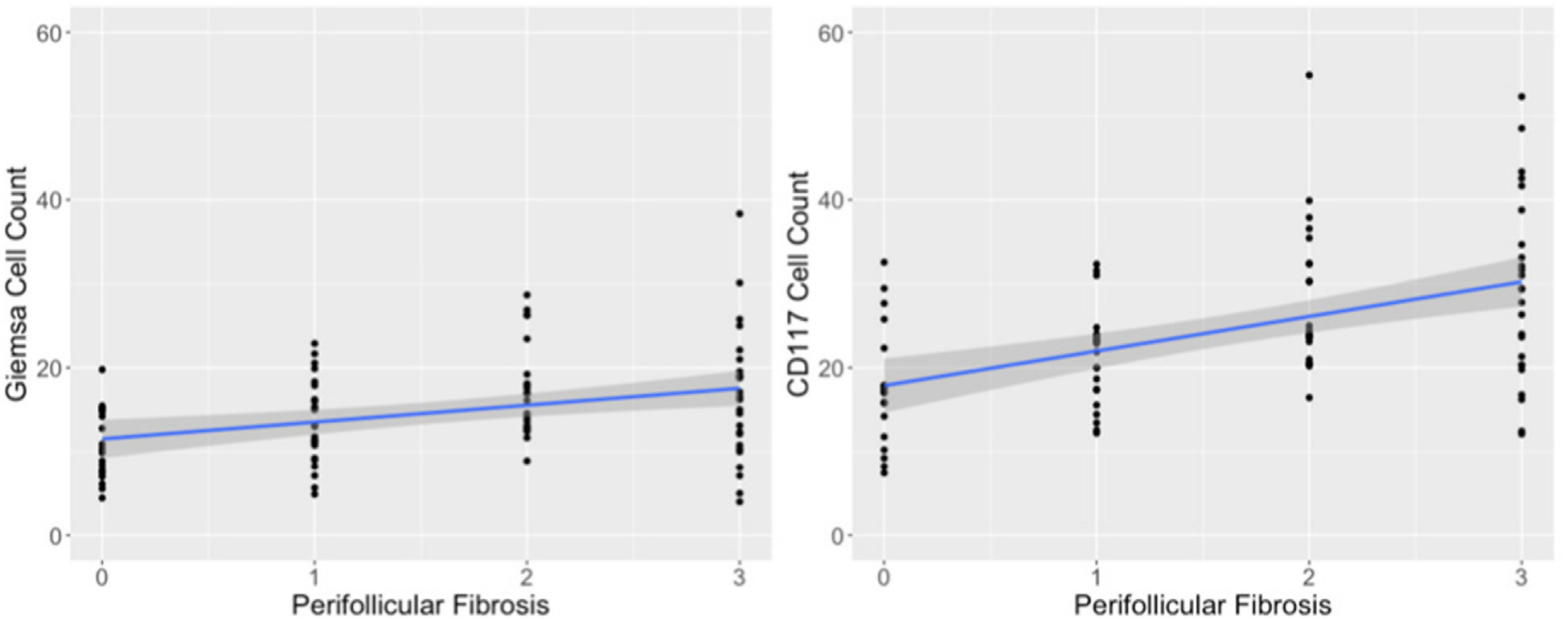
Correlation of central centrifugal cicatricial alopecia mast cell counts with Giemsa stain and immunoperoxidase stain for CD117 with the degree of perifollicular fibrosis. Both were statistically significant (Giemsa *P* = .001, CD117 *P* < .001).

The number of MCs correlated with the degree of perifollicular fibrosis for both stains (Giemsa: *t*_85_ = 3.4038, *P* = .001, Fig 5; CD117: *t*_85_ = 4.9528, *P* < .001, Fig 5). The number of MCs did not correlate with the total number of HFs, the number of terminal HFs, or the degree of patient symptoms for either stain. For CD117 there was a positive correlation between MC count and duration of disease, as well as MC count and disease severity, but neither achieved statistical significance. Both MC count (Giemsa: *P* < .01; CD117: *P* < .001) and degree of inflammation (Giemsa: *P* < .01; CD117: *P* < .01) had concurrent statistically significant positive associations to perifollicular fibrosis in a multivariate analysis controlling for each along with disease duration, patient age, and disease severity.

## Discussion

In our study, significantly more MCs were identified using immunoperoxidase staining for CD117 rather than Giemsa, a finding in keeping with previous studies. Grace et al^8^ found significantly more MCs using an immunoperoxidase stain for tryptase in comparison to Giemsa, and Almodóvar-Real et al^16^ found more MCs with immunoperoxidase stain for tryptase than toluidine blue. Standard histochemical stains for MCs such as Giemsa and toluidine blue induce metachromatic staining, which can produce inconsistent results depending on pH and granule content.^17^ Additionally, the change in color achieved by such staining can be difficult to appreciate depending on the staining intensity. Although it appears that immunoperoxidase staining for CD117 is more sensitive than Giemsa stain for the presence of MCs, this antibody identifies the extracellular domain of transmembrane tyrosine kinase receptor c-Kit. This is not specific for MCs and can also be found in melanocytes and hematopoietic cells,^17^ thus there is the theoretic possibility that other cells may have been inadvertently counted, although the presence of these cells would be highly unlikely.

More MCs were found in specimens that contained the isthmus, as opposed to those containing solely the infundibulum. This is not surprising as inflammation in CCCA, when present, is typically at the upper isthmus and lower infundibulum,^18^ although these levels are sometimes considered together in the literature. It is noteworthy that the number of MCs found on staining for CD117 correlated with the degree of inflammation. Of the 87 patients studied, 60 (69%) had mild or sparse inflammation, either perifollicularly or perivascularly. This is in keeping with the finding that 77% of CCCA biopsies cut in horizontal section had little to no inflammation.^6^ However, when only patients with CCCA whose biopsies included the isthmus were considered, the proportion of biopsies with moderate or heavy inflammation increased from 31% to 53%. This direct correlation of inflammation and MC number differs from the findings in other lymphocytic scarring alopecias (discoid lupus erythematosus and LPP), where a decrease in MCs was observed with increasing lymphocytic inflammation, albeit insignificant.^13^

We also found that in our cohort of patients with CCCA, the number of MCs counted with both Giemsa and immunoperoxidase staining for CD117 was positively associated with the degree of perifollicular fibrosis, both independently and after controlling for other factors. The same was true for the degree of inflammation. This was an expected finding as immune cells, including MCs, are known to play a role in mediating fibrosis in physiologic processes such as wound healing^19^ and in fibrotic diseases such as systemic sclerosis.^20^ In 2003, a patient subsequently diagnosed with systemic mastocytosis presented with a scarring alopecia that contained increased MCs in the inflammatory infiltrate.^21^ Patients with primary scarring alopecia, including both discoid lupus erythematosus and LPP, have been reported to have increased MCs in their inflammatory cell infiltrate.^11^^,^^16^ Hobo et al^11^ used immunohistochemical staining with tryptase to determine that in their 20 patients with LPP, MCs were increased in number compared with control skin. Dual immunofluorescence staining revealed that these MCs stained for IL-23 and IL-17A, the latter of which is known to be profibrotic.^11^ Although Shahidi-Dadras et al^13^ found a statistically significant increase in MCs using Giemsa stain in LPP compared with discoid lupus erythematosus, there was no correlation with MC number or location with the degree of perifollicular fibrosis. Further studies are needed to determine whether it is predominantly MCs, lymphocytes, or both, as the data suggest, that contribute to the formation of perifollicular fibrosis in CCCA.

This study is among the first to find a correlation between MC number and the degree of inflammation with the degree of perifollicular fibrosis in CCCA, and in scarring alopecia in general. Of note, MC number with either stain did not correlate with the total number of HFs or the number of terminal HFs, the latter felt to be better indicator of how advanced the disease was at the time of biopsy. There was a correlation of MC count with immunoperoxidase staining for CD117 with duration of disease as well as disease severity, although it did not achieve statistical significance. One possible explanation for this is that HF number could be more of a reflection on the biopsy site as opposed to disease severity or duration. Additionally, a recent gene expression profiling study suggested that the clinical variability in extent of CCCA may be genetically determined and not dependent on disease duration, although confirmatory biopsies were only obtained in patients with early disease.^22^ Macrophage-mediated and not MC-mediated processes were implicated.

Our study had several limitations, the most significant of which was that the level of staining was dependent on the tissue remaining in the block. Some biopsies were not included due to insufficient tissue, and others contained only the level of the infundibulum. No characterization of MCs in terms of morphology, the presence of degranulation, or cytokine signature was attempted; this may be a consideration for future studies. In addition, the number of lymphocytes was not counted. The finding that the extent of perifollicular fibrosis correlated with the number of MCs with both Giemsa and CCCA is novel and suggests that MCs, in addition to lymphocytic inflammation, may be a therapeutic target in CCCA. A prospective study on CCCA specimens processed longitudinally incorporating histopathology, immunopathology, and further gene expression profiling would be of great benefit to further understand the role of MCs in this condition.

## Conflict of interest

None disclosed.
